# “It’s Like Having a Map”: An Exploration of Participating Pet Owners’ Expectations of Using Telemedicine to Access Emergency Veterinary Care

**DOI:** 10.3390/vetsci12050460

**Published:** 2025-05-12

**Authors:** Rosalie Fortin-Choquette, Jason B. Coe, Cathy A. Bauman, Lori M. Teller

**Affiliations:** 1 Department of Population Medicine, Ontario Veterinary College, Guelph, ON N1G 2W1, Canada; jcoe@uoguelph.ca (J.B.C.); cbauman@uoguelph.ca (C.A.B.); 2 College of Veterinary Medicine and Biosciences, Texas A&M University, College Station, TX 77843, USA; lteller@cvm.tamu.edu

**Keywords:** telemedicine, access to veterinary care, pet owners, semi-structured interviews, thematic analysis

## Abstract

(1) Background: Telemedicine is increasingly recognized as a potential way to help overcome barriers to accessing veterinary care. This study explored pet owners’ perspectives on telemedicine services in situation they viewed as veterinary emergencies, focusing on both their access-to-care challenges and their expectations of such services. (2) Methods: Semi-structured, one-on-one interviews were conducted with 18 pet owners in Ontario, Canada. All participants were unable to access in-person care, leading them to a veterinary telemedicine service. Interviews were supplemented by an online questionnaire to collect demographic information. Thematic analysis was conducted on all interview transcripts. (3) Results: Participants’ expectations of telemedicine were largely informed by their uncertainty about their pets’ health in emergency situations. Anxiety was common, leading them to seek telemedicine for reassurance. Participants expected advice and guidance, viewing the process as collaborative. While acknowledging the benefits of telemedicine in their situation, participants also acknowledged the inherent limitations of this type of service, articulating a general preference for in-person veterinary care. (4) Conclusions: When in-person care is unavailable, telemedicine can offer pet owners valuable guidance, clarity, and comfort, although many pet owners may continue to prefer face-to-face consultations for comprehensive care.

## Simple Summary

Telemedicine is sometimes thought to increase access to veterinary care in certain contexts, such as when in-person care is inaccessible. To gain a better understanding of pet owners’ expectations of telemedicine services when in-person care cannot be accessed, interviews were conducted with telemedicine veterinary clients. The interviews revealed that participants experienced feelings of uncertainty and anxiety. Those feelings seemed to stem from the veterinary emergency they believed to be experiencing and the barriers they faced in accessing in-person care. Participants expressed value in the guidance, clarity, and comfort they gained from telemedicine, given their situation.

## 1. Introduction

Access to veterinary care is a recognized problem in North America. A survey of 5652 pet owners across the United States indicated that 25% of those surveyed had experienced barriers to accessing veterinary care [1]. Likewise, a recent survey representative of Canadian pet owners identified that 18% could not obtain preventive care for their pet in the past year, 12% could not obtain sick care for their pet, and 8% could not access emergency care [2]. In light of these prevalent access-to-care issues, which may be caused by socio-economic, geographic-, demographic- or knowledge-based barriers [3,4], telemedicine has been identified by some professionals as a potential solution for optimizing the accessibility of veterinary care [5,6,7]. However, whether telemedicine platforms are able to meet veterinary clients’ needs and expectations remains a debated issue within the profession [7,8]. It is important to note that the term “virtual care” refers to a broader definition, which includes a wide range of healthcare services delivered remotely. In contrast, the term “telemedicine”, which is predominantly used in this paper, refers more specifically to the remote delivery of advice or treatment based on diagnoses and requires a valid veterinarian–client–patient relationship (VCPR) [9].

Despite existing uncertainty, veterinary telemedicine is increasingly being used by pet owners, with recent market research predicting an approximate growth rate of 20% from 2025 to 2030 [10]. Pet owners’ attitudes towards veterinary virtual care, more broadly, has been studied, with some elements of remote care being linked to satisfaction [11,12] and other elements contributing to a level of reticence towards the service [13]. Specifically, surveyed American cat owners reported being interested in the accessibility benefits of telemedicine, as well as the stress-free option for their pet that is associated with such services [11]. Another survey suggested that pet owners viewed telemedicine services as a favorable adjunct to a traditional, in-person model, citing that telemedicine added educational opportunities and served to “augment, rather than replace, the traditional VCPR” [12]. Conversely, a recent cross-sectional survey reported that pet owners, while open to virtual consultations, preferred face-to-face appointments, and that they were not confident with the communication and relationship-building aspects of remote care [13]. These conflicting findings indicate a need to further explore client attitudes to telemedicine specifically—an exploration which may benefit the profession’s understanding of this manner of delivering veterinary care.

Although virtual care may offer a tool to increase access to veterinary care, there are numerous limitations to its use that have been raised by veterinary professionals, particularly the inability to conduct a physical examination of the patient, which may hinder veterinarians’ ability to reach a diagnosis [8]. Ethical and legal concerns (which include, for example, the inability to establish an in-person VCPR in some cases) related to remote veterinary care are also present within the profession [14]. A previous series of interviews with a sample of veterinarians identified a view among participants that virtual care was “better than nothing” in contexts where it can facilitate accessibility for pet owners [7]. Specifically, interviewed veterinarians agreed that virtual care could be used as a tool to increase access to care but saw limitations in its delivery [7]. To date, however, pet owners’ perspectives of telemedicine, used when in-person care is difficult to access, have not been explored. This knowledge gap raises many questions, including the following: “What are pet owners’ perspectives and expectations of telemedicine when used as a tool to access care in emergency situations, when in-person care is difficult to obtain?” The objective of this study is to provide foundational understanding within this specific context.

## 2. Materials and Methods

This study was approved by the University of Guelph Research Ethics Board (REB# 22-11-023, approved 3 February 2023). The Consolidated Criteria for Reporting Qualitative Research (COREQ) guidelines for interviews and focus groups were followed.

### 2.1. Study Design

Recruitment was a collaborative process with a commercial telemedicine platform that provided virtual veterinary triage services and consultations to support brick-and-mortar practices (by adding this service after hours or when the physical practice is at capacity). Participants were clients of this platform and were able to opt in or out of the study, if they were eligible. At the time of study recruitment, the commercial telemedicine platform was available to veterinary practices and their clients only in Ontario, Canada. In Ontario, veterinarians are permitted to establish a VCPR without an in-person examination of the veterinary patient [15]. The commercial telemedicine platform chosen for this study was not a direct-to-consumer platform. Therefore, sampling from their client base ensured that all participants had attempted to contact a brick-and-mortar practice and had been redirected to the telemedicine service. Thus, all participants had experienced access-to-care issues and obtained telemedicine services for their animal’s care. When clients and patients were redirected to the commercial telemedicine platform, all went through a teletriage process with a Registered Veterinary Technician, and cases that were deemed appropriate for telemedicine consultations were transferred to an online veterinarian.

Recruitment involved convenience sampling of clientele accessing the commercial telemedicine platform. After their telemedicine consultation, clients received a unique link via email to complete the telemedicine service’s standard satisfaction survey. This satisfaction survey was used to recruit participants by displaying information about the present study at the end of the satisfaction survey. Study details were followed by questions related to inclusion and exclusion criteria. To be eligible, participants had to be 18 years of age or over, be the owner or the primary caregiver of the pet who was receiving the telemedicine consultation, and have used the telemedicine service only because they were unable to access in-person care. Participants who indicated interest and were deemed eligible were automatically sent an email with a link to a Microsoft Bookings page (1.5.00), where they could book an interview with the principal investigator (RFC). Upon booking an interview, an online questionnaire was sent to participants using a personalized link, which included a consent form, demographic questions, and questions pertaining to their situation regarding accessing veterinary care, to be completed ahead of their interview date. Interviews were conducted via Microsoft Teams (1.5.00) or by phone from 22 March to 24 April 2023. Incentive to participate was provided via a CAD 25 Amazon gift card, which was sent electronically following the interview.

### 2.2. Questionnaire

The pre-interview online questionnaire was constructed using Qualtrics (Provo, UT, USA). The following demographic information was collected with the questionnaire: gender (woman, man, my gender identity is not listed above, or choose not to respond), age (open text box), country and state/province in which they currently live (open text box), and kind of pet owned (dog(s), cat(s), small mammal(s), reptile(s), amphibian(s), bird(s), and/or other; please specify). Participants were also asked whether they considered their pet to have complex medical needs (yes/no). This item reflected the client’s own assessment, which may or may not have been informed by a prior veterinary diagnosis or discussion.

The following information was collected pertaining to their access-to-veterinary-care situation: if, at the time, they had a regular veterinary hospital for their pet(s) (yes/no). If they selected yes, they were asked to specify the approximate travel time to their regular hospital. If they indicated that they did not have a regular veterinarian, participants were asked to specify the approximate travel time to their nearest veterinary hospital.

### 2.3. Interview Guide

A semi-structured interview guide (Appendix A), constituting seven primary questions and numerous prompts and probes, was formulated based on previous findings regarding veterinarians’ perceptions of virtual-care delivery [7] and the current veterinary literature pertaining to veterinarians’ and pet owners’ perspectives of telemedicine [8,13,14]. The first question explored the participant’s experience relative to accessing veterinary care leading to the interview, and the last question included an example of an access-to-care situation taken from the primary author’s (RFC) personal experience as a pet owner, as a way to add relevance and relatability with respect to access to veterinary care. The anecdotal experience recounted a dog with porcupine quills in its mouth on a statutory holiday, in an area where emergency clinics were several hours away, and the wait time was high. Participants were asked for their opinion on whether telemedicine could have an impact in such situations: “Given your situation, what would happen if, say, your pet had porcupine quills in its mouth? In such an emergency, how would you seek veterinary assistance?”

The interview guide was reviewed and discussed by all authors (RFC, JBC, CAB, and LMT) to ensure relevance, as well as with two pet owners known to the research team and with no scientific background, to ensure clarity. At the time of data collection, the principal author (RFC) was a PhD candidate studying veterinary virtual care, who had previously worked in veterinary clinics, with years of experience as a primary caregiver for pets. RFC, who was also the interviewer, adopted an intentionally curious, dispassionate, and nonpartisan stance throughout the interviews. To ensure integrity of the data collected, the interviewer took notes throughout the interviews and provided participants with an oral summary at the end of each session, a practice which promoted member-checking of the data collected. All interviews were recorded with the Microsoft Teams’ (1.6.00) integrated feature. Recordings were roughly transcribed using the Microsoft Word (16.89.1) transcription function and later reviewed by RFC and a hired transcriber to revise and confirm verbatim transcripts.

### 2.4. Analysis

Reflexive thematic analysis (RTA) was conducted inductively by RFC, guided by the principles articulated by Braun and Clarke [16,17,18,19]. NVivo (1.7.1) was used as a tool to support the organization and management of the data. No predefined coding framework was applied. Instead, coding was open and organic, shaped through iterative and immersive engagement with the transcripts. Themes were not treated as summaries of topics but were constructed as nuanced interpretations that captured underlying patterns of meaning across participants’ accounts. This process involved additional memoing, including observations about participants’ body language and their interaction with their environment, to deepen analytic engagement and support a holistic understanding of the interviews. Reflexivity was central to the approach, with ongoing reflection on the researchers’ positions and influence on the analytic process. Regular discussions between the principal author (RFC) and second author (JBC) helped to critically examine interpretations and enhance the rigor of the analysis.

## 3. Results

### 3.1. Participants

Twenty-one participants were interviewed as part of this study, but three recordings were lost because of technical difficulties during an organizational update to our secure storage area, leaving 18 interviews to be included in the thematic analysis.

From the 18 pet owners interviewed, 17 identified as women and one participant as a man. The youngest participant was 29 years old and the oldest was 71. Seven participants were dog owners, five were cat owners, five owned both cat(s) and dog(s), and one indicated that they owned dog(s), cat(s), bird(s), and goldfish. Five participants indicated that they owned at least one pet with complex medical needs requiring frequent veterinary care. Only two participants did not have a regular veterinary hospital for their pets at the time of the study, and these participants indicated a travel time of 10 and 30 min, respectively, to the nearest clinic. The average travel time for the 16 participants who indicated having a regular veterinary hospital was 27 min (min = 5 min; max = 80 min). Specific details relating to the access-to-care issues faced by participants, leading to their participation in the interview, are summarized in Table 1.

### 3.2. Thematic Analysis

Data saturation, meaning the point at which no new information emerged, was achieved prior to reaching the end of the 18 interviews. In other words, based on the richness and depth of the insights gained from the interviews, we determined that further sampling was not necessary to reach information power [16]. Three overarching themes were revealed through the interviews, relating to telemedicine as a tool to access care: (1) pet owners’ expectations and experiences; (2) the value of telemedicine lies in the contact with the professional; and (3) veterinarian–client communication holds significance when interacting virtually. The overarching theme relating to pet owners’ expectations and experiences was selected for the present paper because of its richness and relevance to the primary research question, which explores pet owners’ perspectives on and expectations of telemedicine when used as a tool to access care in perceived emergency situations. Five subthemes were identified as falling within the main theme of “pet owners’ expectations and experiences”: (1) Anxiety is a common emotion before accessing emergency care via telemedicine. (2) Uncertainty drives expectations of telemedicine. (3) Guidance is expected. (3) Telemedicine is a collaborative process. (5) Face-to-face care is preferred, yet telemedicine can be an option.

#### 3.2.1. Anxiety Is a Common Emotion Before Accessing Emergency Care via Telemedicine

Almost all participants described a feeling of anxiety when they had sought help via telemedicine. This anxiety appeared to be linked to participants’ relationship with their pet and concern for their pet’s health, which was described by many participants as being exacerbated by the access-to-care issue that they were facing.

*“Like… like, there’s nothing worse than being a pet owner and having an animal that is sick and not knowing what to do to help them and not being able to contact somebody. Like, that’s… that’s a horrible feeling.”* (P213w)

The relationship between participants and their pets was noticeable throughout the interviews. Responding pet owners’ affection for their pet was obvious. For example, one participant, with their camera on, could be seen petting their dog on their lap while being interviewed. Another participant was feeding their cat and talking to them throughout their interview. A few participants expressed their affection for their pet explicitly by referring to them as their “child” or their “kid”.

*“Yeah, I mean like as millennials with like no kids… like our pets are our kids.”* (P222x)

The anxiety felt by participants seemed to impact their perception of the gravity of the issue. For example, after their telemedicine appointment, a few participants mentioned that what they had considered to be an emergency (prior to the appointment) may have not, in fact, been one. This suggested that their state of anxiety had altered their judgment regarding their animal’s health status. These pet owners stated or implied that their stress was alleviated by the calm conversation they had with the veterinary professional with whom they had connected through the telemedicine platform. Participants expressed that the calm conversation helped them to gain perspective and to evaluate the situation in a more objective way. One participant described the conversation to be “simple and clear and short and sweet”, (P219w) which helped them to remain calm.

*“She was able to really parse and understand and say, ‘let me understand the sequence’ and got me to kind of slow down a little bit. And very early on, she said, I don’t think we’re dealing with anything terribly serious here, which helped me like, OK, all right, good, take a breath now.”* (P220x)

A handful of participants expressed that before accessing the telemedicine service, they felt as if there was no one available to help them. They elaborated by expressing that while accessing the service, the veterinarian’s caring demeanor and appreciation of their stressful situation led them to feel heard, as the veterinarian seemed engaged in the conversation.

*“I mean, there just happened to be somebody on the phone, who bothered listening… that meant the whole world to me.”* (P216w)

Another participant described that collaborating with the veterinarian, who gave them “clear instructions on what they wanted [them] to do” (P220x), helped lower their stress level, so they no longer felt overwhelmed by the tasks at hand—a view that was observed across the sample in general. Participants indicated that by understanding “the critical factors”, (P220x) as well as by gaining an understanding of “how much of an emergency [their pet] was in”, (P219w) they were able to reduce their anxiety.

*“How serious is this? I needed an answer to that question. How serious is this? Because I was on the end of ‘oh my God, he’s bleeding internally’, just like my last dog who ended up dying. So, it was very, you know this… to me, it was an emergency until somebody could say it was not an emergency. And I got that answer.”* (P220x)

#### 3.2.2. Uncertainty Drives Expectations of Telemedicine

Throughout the interviews, uncertainty emerged as an underlying force behind many participants’ decision to use telemedicine, and behind their expectations of the outcome from the consultation. Before accessing the telemedicine service, almost all participants reported not knowing what was wrong with their pet and not knowing what to do to help them, which they described as making them feel “helpless”, “anxious”, “hesitant”, “worried”, or “panicked.” Several participants thus described using virtual-care services with an expectation of resolving uncertainty by gaining guidance and professional advice.

*“But just having that extra bit of clarity and quite frankly, just having someone else there to be like ‘this isn’t as bad as you think’, or ‘hey man, you know, this is pretty bad, but like, this is how we’re going to deal with it’, right? Because we [pet owners] got no clue.”* (P212w)

Additionally, when describing their experience, several participants expressed uncertainty regarding the veterinary healthcare system as a whole, as all interviewees were unable to access in-person care during their emergency, regardless of whether they had a regular veterinarian. Some, for instance, stated that the emergency clinic near them had a backlog of patients, and that they did not know when or if they should drive there. Other participants had different access issues, as provided in Table 1. After trying and failing to access in-person care, participating pet owners described becoming uncertain of how to gain access to veterinary care until ultimately discovering the option of a telemedicine appointment. One participant, after having called their regular veterinary clinic and multiple clinics in neighboring towns, described feeling panicked and uncertain of what to do:

*“You know, I was desperate. So, I called back our regular clinic and I asked what I could do and they, they gave me the phone number for the telemedicine […] this was the first time that I’ve ever experienced such panic really.”* (P219w)

Uncertainty was also expressed by several participants in relation to using a telemedicine service for the first time, stating that they did not have any expectations of the outcome of their consultation due to lack of previous experience.

*“Um, I really didn’t have any expectations because I didn’t—I hadn’t even heard of this service, and I didn’t know what to expect. So, I was a little bit hesitant, hesitant at first because I thought it wasn’t going to be much help, but it, it, it turned out to be a wonderful positive experience and I will certainly use the service again.”* (P213w)

In relation to this, a few participants described that their uncertainty and in turn anxiety were exacerbated by being redirected to an unfamiliar, virtual system. This uncertainty led them to express the expectation that telemedicine platforms should be simple and easy to use.

*“Now it might have said that on the screen somewhere else, but I was in such a state of high anxiety […] It was one of those weird, technical… you know, the instructions need to be absolutely clear for people who are panicking.”* (P220x)

#### 3.2.3. Guidance Is Expected

When asked during their interview for their explicit expectations of the telemedicine service before accessing it, most participants identified an expectation to receive “any help” possible. For example, several participants described a clear expectation for gaining professional advice to overcome the uncertainty they were experiencing from their situation.

*“Um, I just- I… yeah, I guess guidance is what I wanted. I wanted to know how much of an emergency he was in […] I didn’t know if I was right with my intuition, and I just wanted to know if I should be driving. Like if there was a place for me to go with my puppy over, over a weekend or if there’s anything I could do to try to help him out.”* (P219w)

Similarly, some participants mentioned expecting the telemedicine consultation to “jump start” the process or to be used as a “screening tool”, with several participating pet owners viewing telemedicine to be a temporary option to initiate help or provide guidance until obtaining in-person care.

*“So, the metaphor that comes to mind to me is I’m on a journey with my dog and my destination is his wellness … him getting better. And I’m heading out into the wilderness. I know it’s out there someplace. Right now, it’s at the emergency clinic because that’s where I’m trying to get to. And on the way I find a vet who acts as a signpost. It’s like having a map.”* (P220x)

For a couple of participants, their expectations of the telemedicine service went beyond guidance, with specific assumptions of confirming a diagnosis and receiving a prescription. These participants described having a good idea of what was happening to their pet before accessing the telemedicine service, which they attributed to similar previous experiences. For these participants, uncertainty regarding their pet’s health status appeared to be non-existent. Instead, these participants conveyed clear expectations for the outcome of the consultation, as illustrated by one individual’s description of calling the telemedicine service for a “pretty simple issue”. When asked if they expected a prescription, this participant answered as follows:

*“I did. This is an issue that my cat has had in the past and previous medication she had worked for it as well. I saw in the instructions prior to booking the appointment that if medication was to be prescribed an extra fee would be charged. So, I yeah, I knew it was possible.”* (P204v)

#### 3.2.4. Telemedicine Is a Collaborative Process

Collaboration between the veterinarian and pet owner was mentioned repeatedly throughout the interviews, being described by all participating pet owners as an inherent part of their telemedicine appointment. When relaying a detailed description of their consultation, all participants revealed that they had helped the veterinarian perform certain tasks (such as checking the color of their pet’s gums). Reflecting on their virtual consultation, one participant said:

*“Like you’re going to be their hands and they’re going to be the brain and they’re going to say do this, do that. Working to fix the problem.”* (P207v)

Reinforcing the collaborative, team-based approach to care participants experienced, they often used the word “we” when describing their teleconsultation:

*“We did like—as much of the physical check-up as possible. So, I was able to get her eyes checked out […], we checked her breathing. We checked like her mouth and her nose and everything.”* (P204v)

In some cases, the team-based approach was raised explicitly by pet owners who conveyed a feeling of satisfaction as a result of the collaboration. One participant, for example, expressed a feeling of empowerment and meaning from being involved in their pet’s care:

*“You know, I handle my dog all the time. I clean up his poo. Handling him and… and helping assess him was very meaningful and powerful for me.”* (P220x)

Some participants also indicated that the collaboration aspect of their telemedicine consultation offered them a learning opportunity. The need for collaboration and the team-based approach taken by the veterinarian allowed participants to broaden their experience as pet owners.

*“Like [with] an in-person consultation, it’s more of like the doctor, the vet is taking care of your dog. They know what to look out for, um… [in person] is less of a learning experience for us.”* (P222x)

Furthermore, almost all participants mentioned that the veterinarian coached them on how to perform specific tasks throughout the telemedicine appointment, most often in relation to performing a physical exam. The importance of the veterinarian’s communication skills, specifically in providing clear directions, was commonly highlighted by participants when talking about being coached, with participants noting specific wording and instructions that were given to them.

*“And then like palpate his belly: does it feel hard? And then she was… she was making a commentary, like: OK, it doesn’t look like it bothered him.”* (P200v)

Several participants identified feeling comforted by the collaborative aspect of their telemedicine consultation, describing that it allowed them to feel they had regained control of the situation in which they had previously felt powerless.

*“Being able to coach me over video chat, to understand whether this was life threatening or not… that really helped lower my anxiety. Now I’m back in control. I know what I’m doing, etc.”* (P220x)

Although some participants described feelings of comfort or satisfaction with the team-based approach necessary to conduct a remote consultation, one pet owner raised a feeling of uneasiness brought on by this added responsibility, but ultimately concluded that it was “better than nothing” when in-person care was not available.

*“I just didn’t know completely what to look for. But she did guide me through a lot of it. So, it was OK. It was fine. It’s better than nothing.”* (P215w)

A couple of other participants mentioned that although it did not affect them personally, they could see the collaborative aspect of telemedicine being harder for pet owners who did not feel as bonded with their pet, or who did not have extensive experience in handling them. Indeed, some participants mentioned that the outcome of their telemedicine consultation was impacted by their personal level of comfort during the appointment, as well as their experience as a pet owner. It was not uncommon for participants to indicate that their skills as a pet owner/handler were utilized as part of the collaborative process. One participant specifically acknowledged the impact of a client’s role on the veterinarian’s assessment:

*“Um, but I think … it is a bit of a distributed responsibility at that point, because in case you don’t look [at your pet] very well, or you don’t… or you’re not sure, then basically the doctor is making their assessment based on your information.”* (P218w)

#### 3.2.5. Face-to-Face Is Still Preferred, Yet Telemedicine Can Be an Option

Almost all participants indicated a preference for face-to-face interaction with their veterinarian and viewed telemedicine to be supplemental and reserved for certain contexts, acknowledging that telemedicine comes with limitations. While several participants expressed gratitude throughout their interviews for the help they received, many pet owners clearly expressed that they did not view telemedicine as a replacement for in-person veterinary care. One participant also warned that although they were satisfied with the service, telemedicine should not become a “quick fix” to replace in-person interactions. One participant summed up the various views, acknowledging that telemedicine could become a choice for pet owners depending on the circumstances, and that despite its limitations, telemedicine has some benefits.

*“There will be a time and a place when [telemedicine] is the first choice for me, and then there’ll be a time and a place when it’s the second choice, because I can’t get my first choice, which is to go to see my vet, you know? But if something comes up like I might, I might literally use [telemedicine] as my first choice. When again, I want that advice, I want that education, um I want a certain kind of opinion.”* (P220x)

## 4. Discussion

Results from this set of interviews suggest that when unable to access in-person veterinary care during a perceived emergency, pet owners seeking care via telemedicine have specific expectations for their consultation. Notably, participating pet owners tended to expect specific advice, general guidance, and reassurance from their telemedicine appointment. This expectation seemed to be driven by uncertainty and anxiety caused by the inability to access in-person veterinary care, their pet’s unknown health status, and the unfamiliar process associated with accessing telemedicine. In turn, the uncertainty and anxiety appeared to impact participants’ emotional state leading up to their virtual consultation.

All participants in the present study were in a situation where access to in-person care was not possible, and results suggest that this specific situation exacerbated their feeling of uncertainty already associated with their animal’s health status. In other words, in addition to navigating a perceived veterinary emergency with their pet, participants felt uncertain of how to access veterinary care. In human medicine, illness uncertainty has been associated with psychological distress, as well as cognitive stressors, including a sense of losing control and a sense of doubt [20], which were feelings described by many participants in the present study. In the human medical literature, illness uncertainty comprises four factors: ambiguity regarding the state of the illness, complexity of the treatment and the healthcare system, lack of information about the diagnosis or the severity of the illness, and unpredictability [21]. Most participants in the present study expressed ambiguity in relation to the health status of their pet, as well as a lack of information about the veterinary healthcare system (which led them to telemedicine—an unfamiliar channel for many of the participants). It should thus not be surprising that results of the present study suggest that clients seeking veterinary care on telemedicine platforms in cases of perceived emergencies may experience a strong sense of illness uncertainty. In a study involving human patients with a chronic illness seeking emergency care, participant interviews identified fear as the driver of their emergency visits and indicated that this fear was linked to uncertainty in relation to their symptoms [22]. Uncertainty has indeed been found to be closely related to anxiety and fear, and in the veterinary realm, it has been suggested that it is important for veterinarians to give specific consideration to it by adopting, namely, an empathic and compassionate approach [23]. In line with these recommendations, some participants of the present study identified their veterinarian’s caring and calm demeanor as a factor that alleviated their anxiety associated with their current situation. It is thus likely important that veterinarians practicing via telemedicine—especially during a perceived emergency where the client and patient were not able to access other care—consider the role of illness uncertainty and a client’s associated emotional state in their approach to the interaction.

Indeed, results of the present study suggest that illness uncertainty and the resulting anxiety experienced by participants impacted their expectations when seeking help via telemedicine. The results highlight that participants’ expectations of telemedicine services lie in the specific advice and the general guidance that can be provided by a veterinary professional at a time when a client may feel uncertain, out of control, and/or powerless. Reflecting these insights, some veterinary virtual-care experts have made a distinction between treatment-oriented and guidance-oriented models of virtual-care services that can be offered to clients [24]. Treatment-oriented models have been described as predominantly including situations where veterinarians have access to the patient’s record or have performed a previous, in-person, physical examination, whereas guidance-oriented models are described as offering relief in cases of “uncertainty” and hold value by offering to empower pet owners with knowledge [24]. In the context of the present study, where participants were experiencing a perceived or real emergency and could not access in-person care, guidance appeared to hold genuine value by empowering them with knowledge and greater clarity. As such, offering guidance-oriented models of telemedicine, specifically in emergency contexts where access to care is otherwise not available, is likely to hold value for clients.

In fact, pet owners’ expectations with regard to the outcome of their telemedicine consultation were explored as part of this series of interviews, with the outcomes of guidance and advice being identified as reducing anxiety for participants. Although there is only sparse research focusing on veterinary clients’ expectations, communication preferences, and perspectives of telemedicine, this subject has been explored to a greater extent with human patients. Experts in human medicine have noted that specific strategies should be employed to ensure patient-centered care (which relates to relationship-centered care in veterinary medicine, as per Stoewen et al. [23]) is provided during telemedicine consultations [25]. As such, to facilitate a relationship-centered approach between veterinarian and client, specific strategies could be employed, such as discussing expectations at the onset of telemedicine consultations. However, it is to be noted that previous research in veterinary medicine found that veterinarians uncommonly solicit clients’ concerns at the beginning of in-person appointments [26]. The study reported that when veterinarians did not solicit concerns at the beginning, they were four times more likely to be presented with a client concern in the final minutes of the appointment, suggesting that only a subset of client expectations or concerns were identified upfront [26]. Therefore, as most participating pet owners involved in this study expressed experiencing anxiety, it would behoove veterinary professionals to pay specific attention to establishing a client’s expectations and concerns at the beginning of a telemedicine appointment. As reported by participants of this present study, understanding a client’s current experience and focusing the teleconsultation on relationship-building and collaboration could be used to mitigate anxiety and provide reassurance.

A particular emphasis on the collaborative nature of their telemedicine consultation was highlighted by many participating pet owners, which was often tied to the veterinarian’s communication skills. For participants, collaboration seemed to allow them to regain power in a situation where they felt “powerless”, which helped to alleviate their anxiety about the situation. This finding reinforces what has previously been suggested: that virtual care fosters a team-based approach to care between veterinarian and client [24], and that the collaborative nature likely requires increased consideration with respect to the communication aspect of the consultation. Participants’ emphasis on the need for a collaborative approach during telemedicine appointments is consistent with research examining clients’ expectations during face-to-face interactions, where pet owners have indicated that they want to be involved in their pets’ care [27,28]. This further suggests that a relationship-centered approach is needed to support telemedicine interactions—an approach which has been shown to be associated with client satisfaction and adherence to treatment [29,30]. Although research specific to the communication aspect of telemedicine in veterinary medicine is still sparse, some guidelines have been established within human telemedicine. For example, an emphasis on attentive listening and agenda-setting is recommended when engaging in remote consultations with patients [31]—two elements that have the potential to bring comfort to pet owners (as mentioned by participants in the present study) and to address upfront the feeling of uncertainty identified during this set of interviews. To successfully manage telemedicine consultations in veterinary practice, the findings of the present study suggest that veterinary professionals should consider a collaborative process with clients, supported by the veterinarian’s provision of clear instructions, as well as having a calm and caring demeanor.

Despite their appreciation of the help received virtually when no other help was possible, all participants from this series of interviews expressed a preference for in-person interactions when acknowledging the inherent limitations of telemedicine. Supporting these findings, a previous series of interviews with veterinarians shed light on the notion that virtual care may be used as a tool to increase access to care, yet with some caveats [7]. The inability to perform a physical exam with their own hands, for example, was mentioned by participating veterinarians as an important limitation of remote care [7]. In addition, concerns in relation to misdiagnosis and quality of care offered through virtual care are prevalent among the veterinary profession [8,14,32,33]. The present series of interviews, however, suggests that clients using telemedicine may often be aware of the limitations of remote care. Results also suggest that clients may take these limitations into account with respect to their expectations of the service, especially during a perceived emergency where no other access to care is available. Thus, telemedicine, as expressed by participants in the present study, may provide specific value to veterinary clients and patients as a stopgap before in-person care can be sought, and as a supplementary tool to support a client’s and patient’s veterinary healthcare journey.

## 5. Limitations

Semi-structured interviews were used to gain a deeper understanding of pet owners’ perspectives of telemedicine as a tool to access care. As with all qualitative research, results are not intended to be generalized to all pet owners but instead may provide valuable insight for consideration by veterinary professionals. In order to provide a deeper understanding of what telemedicine can offer veterinary professionals and clients when access to in-person care was an issue, efforts were made to sample pet owners who used telemedicine specifically in this context. Data saturation was reached, supporting the rigor of the findings within the population accessed for this study.

## 6. Conclusions

This set of interviews shed light on participating pet owners’ expectations of telemedicine during emergency contexts (as perceived by the pet owner) where in-person care could not be accessed. The nature of the situation, where access to in-person care was impossible and telemedicine was used as a last resort, was linked to a feeling of uncertainty—a prevalent theme throughout the interviews, related to the medical issue their pet was experiencing, the veterinary healthcare system broadly, and navigating the telemedicine platform itself. The nature of the human–animal bond was notable throughout the interviews and appeared to be associated with the anxiety participants experienced in relation to their pet’s health status, which, in turn, impacted their experience. Clients’ feeling of uncertainty and the resulting anxiety impacted their expectations, as the intention to receive professional advice and general guidance seemed to be the driving force behind users’ choice to obtain veterinary care via telemedicine. The collaborative nature of the telemedicine interactions was identified as having the effect of alleviating the uncertainty and anxiety experienced by participants. Although satisfied with the service, participants acknowledged the inherent limitations of telemedicine, expressing their general preference for face-to-face interactions when such care is accessible.

## Figures and Tables

**Table 1:** Access-to-care issues experienced by participants.

Issues Preventing Participants from Accessing In-Person Care (Some Pet Owners Simultaneously Experienced Multiple Access Issues) and Presenting Concern(s)	Exemplar Participant Quote
After hours/all clinics closed Cat urinating blood Dog “chewed its stitches” (post-op) Dog “very ill” Dog “poisoned with charcoal” Dog vomiting blood	*“There are no vet clinics that do after-hours care in [town] or in [neighboring town] for that matter, which is half an hour drive. So basically, you have to travel over an hour to find after-hours emergency care. If you can get in. Because, because they’re so busy.”* (P211w)
Overloaded clinics Dog with “very bad” ear infection Cat with eye infection Cat urinating blood Dog bloated, acting strangely, vomiting Dog with possible kidney stones Dog lethargic and vomiting	*“We were- basically it was like 10:30 at night. Uh, we were trying to call every emergency vet practically in the [region]. Every single one was literally, had a, a backlog of six hours. Um…some of them were like, don’t even bother coming, umm a, either because they were just so backlogged, or they didn’t have a neurologist on staff.”* (P216w)
“Care deserts” Dog with severe diarrhea Dog was “in a lot of pain” and “in a health crisis”	*“So, I tried calling in the neighboring towns because we only have the one in town and he only comes here once a week because he services…he services a town about an hour away and he comes here on Thursdays.”* Participant called several places within a few hours’ radius and *“nobody was willing to accept an emergency case of a uh, patient pet that was not already one of their patients, I guess.”* (P219w)
Travel advisory Cat with possible urinary infection/blockage Dog “poisoned with charcoal”	*“So, but this incident, so I’d say it was 9:20 on a Saturday night and it was snowing like crazy out. And they told people, do not, you know, one of those lovely snowstorms when you have to go out and they’re telling people: don’t travel if you don’t have to, because it’s dangerous.”* (P210w)
No means of transportation Dog vomiting blood Cat with eye infection	*“So, I live. I live on [street name]. So, it’s a very busy street, there’s lots of construction and the buses don’t really—What am I trying to say? The public transit is difficult. So, getting there, I usually have to walk honestly, and it’s very difficult [with a sick cat].”* (P204v)
Just moved No nearby clinic taking new clients, cat sneezing intensely (owner worried it could be “very serious”)	*“So, I just, uh, immigrated here. I moved to Canada 3 months ago. Um, so I don’t even have a doctor for myself. So, I don’t even have a doctor. I don’t even have a vet for, for my cat.”* (P218w)
Pet with anxiety related to travel/going to vet Cat sneezing intensely	*“He’s a COVID pet, so I adopted him during the pandemic. So, for him, seeing the vet is extremely stressful.”* (P218w)
Dog whelping risky (for animals’ health) to drive long distance with dog in whelp and puppies in car	*“So, it was sort of about 10 at night and I had a dog whelping, and she wasn’t able to whelp the last puppy.”* Telemedicine allowed the owner in this case to *“continue working with my dog without disrupting the process. We didn’t have to put her in a car. And [otherwise] we would have to take in all the puppies with us.”* (P207v)
Pet with mobility issue Dog with severe diarrhea	*“She does have problems getting around. So, I have, I have trouble getting her to the vet. Like, I have problems getting her in the car because I actually have to lift her up and she’s, I mean, she’s on the small side for Rottweiler, but she’s still like 70 lbs, right?”* (P217w)
Pet with transmissible disease/parasite Recently adopted cat with eye infection	*“As soon as they found out she had worms—which she did—[…] we couldn’t get an appointment right away. Most people weren’t willing to even see it because of what she had.”* (P212w)
Pet with behavioral issues Pressing concern, but cannot catch the cat	*“Well, my cat, the rescue cat that I adopted, is a real introvert and I can’t… I’ve been only able to pick her up twice in three months. She’s been abused, scared, [unintelligible] scared so for me to go to the vet is impossible right now until she gets better.”* (P205v)

## Data Availability

The interview transcripts are not publicly available due to ethical constraints; however, all relevant findings are presented in the article.

## Appendix A

### Discussion Guide

Primary Questions
1. You were referred to us because you have had a hard time seeing a veterinarian in person. Tell us about your experience accessing veterinary care that has led you to this interview...
2. Can you describe the virtual consultation you have just had?
3. Let’s talk about the outcome of the telemedicine visit. When you booked this virtual consultation with a veterinarian, what were your expectations?
4. Now that your telemedicine visit is over. What were/are your expectations following the consultation you have just had?
5. What are your thoughts on the communication aspect of a telemedicine consultation?
6. What are your thoughts on paying for a telemedicine consultation?
7. Before we conclude our discussion, let’s look at an example. Given your situation, what would happen if, say, your pet had porcupine quills in its mouth? In such an emergency, how would you seek veterinary assistance?
